# The Intersectionality of Sex and Race in the Relationship Between Posttraumatic Stress Disorder and Cardiovascular Disease: A Scoping Review

**DOI:** 10.3389/phrs.2023.1605302

**Published:** 2023-06-27

**Authors:** Lauren D. Hunter, Tara Boer, Leia Y. Saltzman

**Affiliations:** ^1^ Tulane Center for Aging and Department of Medicine, Tulane University, New Orleans, LA, United States; ^2^ Tulane School of Social Work, Tulane University, New Orleans, LA, United States

**Keywords:** cardiovascular disease, scoping review, sex, race, posttraumatic stress disorder

## Abstract

**Objectives:** Posttraumatic stress disorder (PTSD) has been linked with cardiovascular disease (CVD), suggesting a risk for negative health outcomes among individuals with PTSD. This review synthesizes the temporal relationship between PTSD and CVD and highlights the intersection of sex and race.

**Methods:** Covidence was used to systematically review the literature published between 1980 and 2020.

**Results:** 176 studies were extracted. 68 (38.64%) of the studies were a predominantly male sample. 31 studies (17.61%) were a predominantly female sample. Most reported participants of both sexes (*n* = 72; 40.91%) and only 5 (2.84%) did not report respondent sex. No studies reported transgender participants. 110 (62.5%) studies reported racial and ethnic diversity in their study population, 18 (10.22%) described a completely or predominantly white sample, and 48 (27.27%) did not report race or ethnicity of their study population.

**Conclusion:** A compelling number of studies did not identify sex differences in the link between PTSD and CVD or failed to report race and ethnicity. Investigating sex, race, ethnicity, and the temporal relationship between PTSD and CVD are promising avenues for future research.

## Introduction

Data from the National Health and Nutrition Examination Survey (NHANES) between 2015 and 2018 suggest that the prevalence of cardiovascular disease (CVD) in adults was 49.2% [1]. CVD is also the leading cause of death among women in the United States [2]. Significant progress has been made in identifying the *biological* factors that elevate the risk of CVD among women [3–5]. Yet, the literature remains less comprehensive regarding the *psychosocial* factors that contribute to the sex differences in CVD. In addition to women, non-Hispanic Black, Hispanic, and Asian adults are more likely to be diagnosed or die from heart disease when compared to their non-Hispanic White counterparts [6]. Few studies have specifically focused on the psychosocial risk and protective factors among racially diverse populations.

Approximately 60% of men and 50% of women experience at least one trauma in their lives [7–9]. A review of the literature by Hatch and Dohrenwend [10] found that traumatic and other stressful events are more frequent in racial and ethnic minorities. Posttraumatic stress disorder (PTSD) is characterized as a pervasive and chronic mental health condition, in which an individual experiences lasting adverse psychological and physiological effects associated with a traumatic event they experienced [11]. Women are twice as likely to meet the criteria for PTSD despite having less exposure to traumatic events [12]. Additionally, Black survivors (8.7%) are also more likely than Whites (7.4%) to receive a diagnosis of PTSD after a traumatic event [9, 13–17]. PTSD has been shown to have a relationship with CVD events [18] and early age heart disease mortality [19]. Additionally, adverse childhood experiences have shown a graded relationship with ischemic heart disease [20]. A meta-analytic review by Edmondson et al [21] found PTSD to be independently associated with the risk for the development of coronary heart disease.

Given the preliminary evidence that PTSD is related to later CVD, and the parallel trends regarding the elevated risk for women and racial and ethnic minorities to experience traumatic events and be diagnosed with PTSD, we hypothesize that PTSD may be more than a risk factor for CVD but an important mediator in explaining the sex and race differences observed in rates of CVD. The current study reviews the state of the literature to identify trends and gaps in understanding the documented relationships between sex, race, PTSD, and cardiovascular disease.

### PTSD

The current definition of PTSD outlined in the current Diagnostic Statistical Manual of Mental Disorders (DSM-V) requires that a person must experience a psychologically distressing event that produces feelings of intense fear and helplessness (i.e., a trauma) [11]. Notable symptoms of PTSD include hyperarousal, intrusive thoughts and memories, avoidance of reminders and activities associated with the trauma, and sensations of re-experiencing the traumatic event (e.g., via flashbacks and nightmares) [11]. The DSM-V added a fourth criterion to previous versions requiring that a survivor also experience negative cognitions or mood changes [11, 22–25].

Besides sex and race, several other psychosocial factors influence the development of PTSD: trauma type [26], socioeconomic status [1], limited social support [27], relationship of the perpetrator [28], limited coping strategies [29], perceiving the impact of the event to be severe [30], and biological and psychological comorbidies [31]. There are sex differences regarding the trauma type that individuals with PTSD experienced. Specifically, one study reported that men identified combat exposure (57.7%) whereas women disclosed rape (74.4%) and sexual molestation (61.4%) as the traumatic events they lived through prior to being diagnosed with PTSD [9].

Determinants that were related to the development of PTSD include resilience [32, 33], morale [34], physical activity [35], and low attachment dependency and anxiety as measured by the Adult Attachment Scale [36]. Beyond an elevated CVD risk, the higher rates of PTSD among women and BIPOC groups are concerning because PTSD is also a risk factor for the development of other psychological issues, like mood, anxiety, and personality disorders [37, 38] and substance misuse disorders [37, 39].

Broadly, PTSD is associated with overall poor health [40–42]. A meta-analysis by Pacella et al [43] found PTSD to be associated with a high frequency of pain, gastrointestinal complaints, and a greater number of medical complaints overall. Additional studies have found significant associations between PTSD, chronic pain [44], somatization of symptoms [45, 46], hospitalizations [47], type 2 diabetes [39] and metabolic syndrome (MetS) [48]. This suggests that PTSD may be an important early warning sign for the development of chronic and costly health conditions.

### Cardiovascular Health

CVD is an umbrella term used to refer to several conditions that affect the cardiovascular system (e.g., strokes, myocardial infarction, atrial fibrillation, heart murmurs, and coronary artery disease). Several established factors that predict CVD have been linked with a PTSD diagnosis, including high blood pressure and heart rate [49], non-adherence to medications [50], alcohol and nicotine use, use [51, 52], poor sleep quality [53], anger or hostility [54], and lack of physical activity [36]. Biomarkers like autonomic dysregulation (including parasympathetic limitations), heart rate, blood pressure, endothelial function, baroceptor sensitivity, and lipids have also been studied [55].

The association between psychological distress and physiological illness can be partially explained by the Diathesis Stress Model [56]. The Diathesis Stress Model suggests that humans are born with biological factors that place them at higher risk for the development and onset of various diseases [57]. McKever and Huff [56] applied the diathesis stress model to PTSD and described three pathways that Mutually influence each other in the development of PTSD: a) residual stress or long-term trauma (i.e., the primary mechanism); b) ecological factors (e.g., relationships, support systems, and coping mechanisms); c) and biological factors (e.g., genetics, neurological composition, inherited genes) [56]. Most notable is the relationship between traumatic stress and elevated levels of hypothalamic pituitary adrenal axis (HPA) activation and cortisol [4]. High cortisol levels have been shown to negatively impact blood pressure, insulin levels, and cholesterol and are therefore influential in healthy heart functioning [58–60].

While components of the relationship between PTSD and CVD have been elucidated in the literature, there is a dearth of research investigating PTSD as a potential mediator of the relationship between sex, race, and CVD. The aims of the current study are threefold: to identify trends in the literature regarding the relationships between sex, race, posttraumatic stress, and CVD; highlight disparities in current knowledge; and suggest recommendations for the next steps in the study of posttraumatic stress and cardiovascular health.

## Methods

### Study Identification and Selection

Literature searches were conducted using Academic Search Complete (ASC), Medline, APA Psych Info, Embase, Psychology and Behavioral Science Collection, PTSD Pubs, and PubMed databases. Articles from Medline, APA Psych Info, and Psychology and Behavioral Science Collection were retrieved from the ASC search engine. Keywords associated with concept one, post traumatic stress disorder included “Posttraumatic Stress,” “Post-traumatic Stress,” “post-traumatic stress,” “post traumatic stress,” “PTSD,” and “ptsd.” Keyword searches for concept two, cardiovascular disease, included, “stroke,” “congestive heart failure,” “heart disease,” “cardiac death,” “stress cardiomyopathy,” “cardiac event,” “Cardiovascular Disease,” “CVD,” “Cardiovascular Health,” “Cardio*,” “Myocardial Infarction,” “MI,” “Atrial Fibrillation.” “afib,” “Arrythmia,” “stroke,” “and heart infarction.” Reviewers utilized advanced search options to include “OR” to capture all possible articles including these two concepts limiting findings to articles written in English between 1980 and 2020. Articles included research with humans. The following limiters were further applied within the ASC database via the “Narrow by Subject Thesaurus” function: “post-traumatic stress disorder,” “atherosclerosis,” “blood pressure,” “cerebral ischemia,” “stroke patients,” “ischemia,” “cerebrovascular disease,” and “coronary disease.” Applicable titles and abstracts were uploaded to Covidence, an online systematic review resource that we used to conduct our scoping review.

### Study Inclusion Criteria and Evaluation

Only empirical research articles were included in this review. Accordingly, selected articles were a) published between 1980 and 2020, b) peer-reviewed, c) available in English, d) involved human participants, and e) had research questions that pertained to PTSD and cardiovascular outcomes. Inclusion criterion A was determined based upon the year that PTSD was officially included in the DSM [22]. Specifically, we included studies that examined prevalence (especially by race and gender), interventions, and the temporal relationship, including mechanisms and mediators between PTSD and CVD.

Studies were excluded if a) the research question contained the inverse of the temporal relationship of our aim (i.e., posttraumatic stress as secondary to cardiovascular disease); b) full text was not available; c) the document was a literature review, meta-analysis, editorial, commentary, or letter; and d) there was not a legitimate measurement tool for PTSD (e.g., clinician administered PTSD scale [CAPS], PTSD checklist [PCL], Davidson trauma scale, a record of PTSD diagnosis from a psychiatrist [ICD-9 code], etc.) or CVD. Additionally, we excluded studies that investigated cardiometabolic disease and hypertension as outcomes of PTSD instead of mediators between PTSD and CVD.

### Data Extraction and Analysis

We adopted a conservative approach to our screening process given that our research question involved a specific direction of a relationship and relied on the usage of validated measurement tools for PTSD and cardiovascular health (both of which are difficult to discern based solely on the abstract). Accordingly, we conducted two waves of screening: an initial title and abstract evaluation to ensure that the basic criteria were met followed by a full-text review to analyze the direction of the relation between PTSD and cardiovascular health and to confirm the utilization of appropriate measurement tools.

Two reviewers evaluated the retrieved abstracts, applied the inclusion criteria, and voted on “fitness” for the full-text screening. If a discrepancy occurred between votes, a third reviewer was asked to be a “tie-breaker.” A total of 2,670 references were imported to Covidence, an online systematic review resource, and after duplicates were automatically removed (*n* = 1,186), 1,484 abstracts were screened. 958 abstracts failed to meet preliminary criteria (see above) and 526 studies qualified for full-text review. Covidence tracked our review process via the PRISMA (Preferred Reporting Items for Systematic Reviews and Meta-Analyses) framework [61].

**FIGURE 1:**
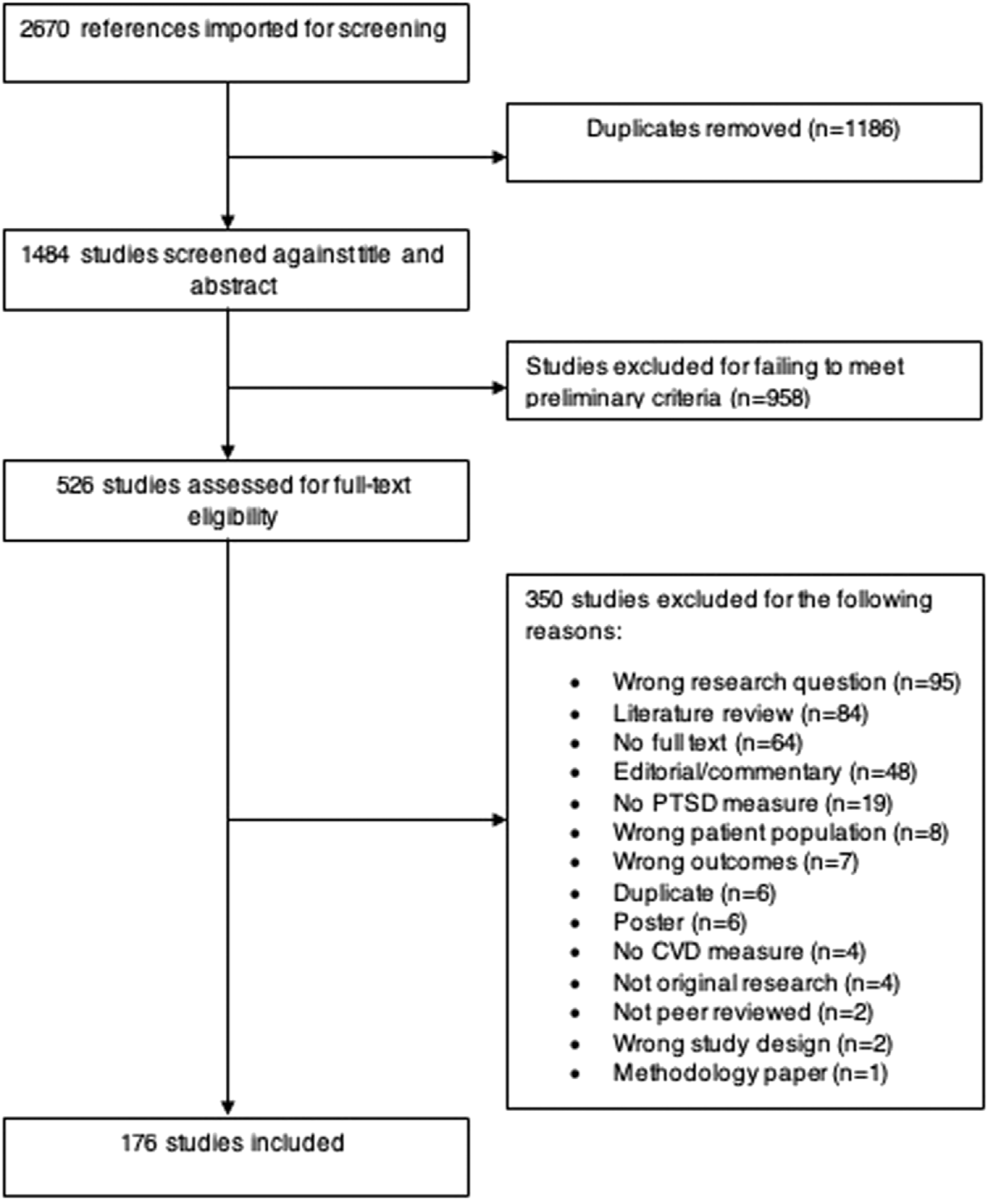
Flow diagram of literature search strategy (United States, 2023).

During the full-text review, 350 studies were further excluded for failing to meet our criteria and a final total of 176 studies were extracted for this scoping review. Cohen’s Kappa for interrater reliability was calculated by Covidence as 0.92 a metric of percent agreement across reviewers. Figure 1 exhibits a flow diagram of our study identification and extraction process.

## Results

### Study Characteristics

One hundred and seventy-six studies were included in this review. Of these 88 (50%) were published in the biomedical field, 70 (39.77%) in social science, and 18 (10.23%) in public health. We classified studies into four kinds of study design longitudinal (*n* = 33; 18.75%); cross-sectional (*n* = 83; 47.16%); intervention (*n* = 14; 7.95%); and other (*n* = 46; 26.13%) which included cohort studies, case-control, and prevalence studies. Of these, only 29 (16.48%) identified potential mediators explaining the relationship between PTSD and CVD outcomes. A range of measurement tools was used to assess both PTSD and cardiovascular outputs (reported in Supplementary Table S1).

### Sex and Race

We further explored the sex, racial, and ethnic breakdown of the studies included in our sample. Sex data was measured categorically when such information was available. No study supplied any details as to whether the participants had the option to report a sex other than male or female (i.e., a non- binary response). Furthermore, gender and sex language appeared interchangeable or conflated in some cases (e.g., “the male gender” [62]), and gender itself was not directly measured in any of the articles under review. The categorical data on race and ethnicity varied across papers. Some papers only displayed “White” vs. “Non-White” statistics with a majority of studies reporting three or more racial categories.

Sixty-eight (38.64%) of the studies were a complete or predominantly, 85% and above, male sample. Thirty-one studies (17.61%) were a complete, or predominantly, 85% and above, female sample. Overarchingly studies reported participants of both sexes (*n* = 72; 40.91%) and only five studies (2.84%) did not report respondent sex. Importantly, no studies reported transgender participants. However, it is impossible to conclude that no transgender individuals participated in these studies since gender was not assessed. It may be the case that transgender individuals did participate in this research but their data was simply not captured by the researchers.

Similar patterns were found regarding racial and ethnic diversity among study participants: 101 studies (57.39%) reported racial and ethnic diversity in their study population, 18 studies (10.22%) described a completely or predominantly white (85% and above) sample, and 57 (32.39%) did not report race or ethnicity of the study population.

A total of 72 (40.91%) studies collected data on both sexes and two or more racial or ethnic categories. Of these, 54 (75%) reported including race and/or sex in interaction analyses. 35 studies (19.88%) collected data on both sexes and at least one or no racial category, of which 20 (57.14%) indicated including sex in their interaction analyses. Lastly, 38 papers (21.59%) showed data on at least one or more racial or ethnic groupings and only one or no sex. Of these, 16 studies (42.10%) reported that race or ethnicity was included in interaction analyses.

## Discussion

Our review was intended to provide an overview of the state of the literature surrounding the relationship between PTSD and CVD. Our initial search resulted in 1,484 articles suggesting the topic has received a fair amount of attention in the literature. Of these, 958 were excluded largely because they specifically focused on PTSD emerging as a secondary consequence of CVD, illness, and treatment (studies were also excluded for having the wrong population, inadequate measurements, etc.). While this relationship is important, it is qualitatively different from PTSD which precipitates cardiovascular disease. The issue of temporality is an important gap that we identified in the literature. While a few studies (*n* = 33; 18.75%) in our review firmly establish a temporal order to the relationship between PTSD and CVD, several *implied* a temporal relationship by examining CVD within a population with PTSD (e.g., veterans). Given the biological and psychosocial capability of a PTSD diagnosis to cause poor health outcomes [41–43, 63] we suggest that this is a key area of exploration that should receive greater attention in the literature using research designs that firmly establish temporal ordering.

While we found greater diversity in study samples than anticipated, only 72 (40.91%) of studies included both sexes, leaving the majority of studies unable to study or identify sex differences regarding PTSD and CVD; both of which are conditions that have demonstrated clear and unique patterns among men and women. Similarly, no studies explored the relationship between PTSD and CVD among transgender participants highlighting the underrepresented nature of transgender and non-binary participants. A more concerning pattern emerged regarding racial and ethnic diversity among study respondents with 32.39% of studies not reporting race and/or ethnicity for study respondents. The lack of racial/ethnic representation in these studies further demonstrates that health disparities begin in research design and that sampling procedures should specifically focus on recruiting racially and ethnically diverse study samples, and that researchers should report demographic characteristics of study participants in their publications.

Only 14 of our final articles (7.95%) were intervention studies. Intervention studies are critical in understanding ways to alleviate not only PTSD symptomology but to improve heart health and decrease risk for CVD among trauma affected populations. Promising therapeutics of note include intranasal oxytocin, respiratory sinus arrhythmia biofeedback, device-guided slow breathing, and variations of exposure therapy. Given the dearth of studies in this arena, future intervention studies should investigate preventative treatment in PTSD patients to reduce risk for CVD as well as expand on retroactive measures. In addition, intervention studies should highlight critical windows of opportunity in which interventions are most effective. Only a small number of studies identified mediation pathways between PTSD and CVD. While there is a plethora of articles that included the word “mediation” in the titles and abstracts, only four studies (2%), to our knowledge, qualified as mediation investigations that were longitudinal and utilized recognized mediation procedures (e.g., Barron and Kenny’s steps for mediation, bootstrapping, etc.). Accordingly, mediation studies may be a promising avenue for those seeking to research the mechanisms between PTSD and CVD.

### Limitations

As with all literature reviews, a limitation of this investigation is the finite time frame of our publication inclusion window. Articles in this review were limited to those published between 1980 and September 2020 which naturally leaves out papers published between September 2020 and the time of this publication. We would be remiss if we did not mention at least a few articles that have been published since the time of our study window. These include Korinek et al [64], who investigated the role of PTSD in war stressors and subsequent CVD in older adults, Seligowski et al [65], who examined sex differences in pharmaceuticals in the relationship between PTSD and CVD, and Gavin et al [66], who explored PTSD as a link between racial discrimination and CVD. We recommend readers who are keen on this topic to use this review as a jumping-off point to the ever-growing field of research in PTSD and CVD. Studies were not limited to those conducted in the US but were restricted to those written in English as all authors were only able to assess work in this language. We note that some important work on this topic may be excluded from our analysis due to this limitation. We did include articles from multinational research teams and global populations and note the measurement tools used to assess our outcomes of interest to ensure consistency across regions.

### Implications and Future Work

While PTSD and CVD have independently received a significant amount of attention in the literature, there remain gaps in the investigation of the relationship between the two. This review offers a summary of the state of the literature thus far and proposes areas of expansion for the next phase of research. With the emergence of more advanced technology, remote monitoring methods, and awareness regarding the impact of stress on the biological and psychosocial functioning of survivors this line of inquiry has the potential to highlight race and sex-specific trajectories, identify critical windows of opportunity for early intervention, highlight important mediators that should be the focus of interventions to ameliorate deleterious symptomology, and ultimately prevent disease onset.
