# Supplementary material for: The Intersectionality of Sex and Race in the Relationship Between Posttraumatic Stress Disorder and Cardiovascular Disease: A Scoping Review
Source: Public Health Rev. 2023 Jun 27;44:1605302. doi: 10.3389/phrs.2023.1605302 (PMC10333493; doi:10.3389/phrs.2023.1605302)
Supplement: Supplementary file 1 [file Table1.DOCX]

| Table 1. Summary of Included Studies | | | | | | | |
| --- | --- | --- | --- | --- | --- | --- | --- |
| **#** | **First Author, Year** | **Title** | **Field of Study** | **Study Design** | **Total Participants (% female)** | **PTSD Measure(s)** | **Cardiovascular measure(s)** |
| 1 | Tegler, 2017 | Successful use of closed-loop allostatic neurotechnology for post-traumatic stress symptoms in military personnel: Self-reported and autonomic improvements | Biomedical | Intervention study | 18 (6%) | PCL-M | BP, HRV, blood and saliva biomarkers for inflammation |
| 2 | Keyes, 2013 | Potentially traumatic events and the risk of six physical health conditions in a population-based sample | Social science | Longitudinal design | 1054 (53%) | PCL-C, CAPS | Self-report of cardiovascular symptoms and physician diagnosed cardiovascular disease |
| 3 | Scherrer, 2019 | Combined effect of posttraumatic stress disorder and prescription opioid use on risk of cardiovascular disease | Public health | Longitudinal design | 2861 (14.3%) | ICD-9 code for PTSD on two or more separate visits within the same 12-month period or one inpatient visit. | Incident CVD via ICD-9 codes |
| 4 | Valentine, 2017 | The association of posttraumatic stress disorder and chronic medical conditions by ethnicity | Public health | Cross sectional study | 7437 (57%) | Modified WHM CIDI | Self-report of cardiovascular symptoms, cardiovascular risk factors, and physician diagnosed cardiovascular disease |
| 5 | Moazen-Zadeh, 2016 | Increased blood pressures in Veterans with post-traumatic stress disorder: A case-control study | Social science | Cross sectional study | 100 (0%) | SCID according to the DSM-IV-TR by a military psychiatrist | Brachial BP, medical records of lipid profile |
| 6 | El-Gablawy, 2014 | A longitudinal examination of anxiety disorders and physical health conditions in a nationally representative sample of U.S. older adults | Social science | Longitudinal design | 10409 (55.4%) | AUDADIS-IV | Self-report physician diagnosed cardiovascular disease |
| 7 | Muhtz, 2011 | Effects of chronic posttraumatic stress disorder on metabolic risk, quality of life, and stress hormones in aging former refugee children | Biomedical | Cross sectional study | 50 (64%) | PDS | Serum glucose, total cholesterol, HDL and LDL cholesterol, triglycerides, h-CRP, hemoglobin A1c, and thyrotropin |
| 8 | Andersen, 2010 | Association between posttraumatic stress disorder and primary care provider-diagnosed disease among Iraq and Afghanistan Veterans | Biomedical | Longitudinal design | 4416 (11%) | VA Primary care physician diagnosis according to the DSM IV | Medical records of circulatory diseases |
| 9 | Tucker, 2012 | Emotional stress and heart rate variability measures associated with cardiovascular risk in relocated Katrina survivors | Biomedical | Longitudinal design | 68 (Not reported) | CAPS | HRV |
| 10 | Vidovic, 2009 | Changes in immune and endocrine systems in posttraumatic stress disorder: A prospective study | Biomedical | Longitudinal design | 76 (0%) | ICD-10 code for PTSD | Blood sample for biomarkers of CVD |
| 11 | Boscarino, 2008 | Psychobiologic predictors of disease mortality after psychological trauma | Biomedical | Longitudinal design | 4462 (0%) | Davidson Trauma Scale-PTSD | WBC, RBC, ESR‚ plasma cortisol, DHEA-s, CVD-related death from the National Death Index-Plus |
| 12 | Violanti, 2006 | Police trauma and cardiovascular disease: Association between PTSD symptoms and metabolic syndrome | Public health | Cross sectional study | 101 (40%) | IES | Metabolic syndrome risk factors (waist circumference, BP, HDL cholesterol, triglycerides, glucose levels) |
| 13 | Yoo, 2020 | Abnormal sympathetic neural recruitment patterns and hemodynamic responses to cold pressor test in women with posttraumatic stress disorder | Biomedical | Intervention study | 28 (100%) | CAPS | BP |
| 14 | Holmstrup, 2020 | Sex differences in the association between PTSD symptoms with cardiac autonomic function and subclinical atherosclerotic risk | Biomedical | Cross sectional study | 61 (48%) | PCL | Carotid femoral pulse wave velocity, flow mediated slowing of carotid radial, HRV |
| 15 | Bourassa, 2020 | The impact of exposure therapy on resting heart rate and heart rate reactivity among active-duty soldiers with posttraumatic stress disorder | Biomedical | Intervention study | 104 (6%) | CAPS-IV, PCL-C | Cardiovascular Reactivity (HR) |
| 16 | Smith, 2020 | PTSD in Veterans, couple behavior, and cardiovascular response during marital conflict | Social science | Cross sectional study | 64 couples (50%) | CAPS | BP, HR, PEP |
| 17 | Rosman, 2019 | Posttraumatic stress disorder and risk for early incident atrial fibrillation: A prospective cohort study of 1.1 million young adults | Public health | Longitudinal design | 988090 (12.2%) | ICD-9 code recorded during hospitalization or at least during 2 outpatient encounters | Incident CVD via ICD-9 codes |
| 18 | Vance, 2019 | Increased cardiovascular disease risk in Veterans with mental illness | Biomedical | Longitudinal design | 1611378 (94%) | ICD-9 code for PTSD | VA risk score of CVD, incident CVD and CVD-related death via ICD-9 codes |
| 19 | Hoerster, 2019 | PTSD is associated with poor health behavior and greater body mass index through depression, increasing cardiovascular disease and diabetes risk among US Veterans | Biomedical | Cross sectional study | 657 (13.5%) | PCL-M | Self-report of hypertension, CVD, diabetes |
| 20 | de Vries, 2016 | Altered one-carbon metabolism in posttraumatic stress disorder | Social science | Cross sectional study | 94 (70%) | SCID | BMI, blood sample for biomarkers of CVD |
| 21 | Hieda, 2019 | Reduced left ventricular diastolic function in women with posttraumatic stress disorder | Biomedical | Longitudinal design | 14 (100%) | CAPS-5 | Transthoracic echocardiogram |
| 22 | Toczek, 2019 | FDG PET imaging of vascular inflammation in post-traumatic stress disorder: A pilot case-control study | Biomedical | Cross sectional study | 16 (38%) | CAPS-5 | Blood glucose level, lipid panel, creatinine, and inflammatory biomarkers |
| 23 | Fonkoue, 2018 | Elevated resting blood pressure augments autonomic imbalance in posttraumatic stress disorder | Biomedical | Cross sectional study | 33 (18%) | CAPS | HR, BP, respiratory rate, HRV, MSNA, BRS |
| 24 | Fonkoue, 2018 | Acute effects of device-guided slow breathing on sympathetic nerve activity and baroreflex sensitivity in posttraumatic stress disorder | Biomedical | Intervention study | 23 (9%) | CAPS | BP, HR, BRS, MNSA |
| 25 | Akosile, 2018 | PTSD symptoms associated with myocardial infarction: Practical clinical implications | Social science | Cross sectional study | 277 (0%) | CAPS-5 | Comprehensive CVD history |
| 26 | Tudor, 2018 | Genetic variants of the brain-derived neurotrophic factor and metabolic indices in Veterans with posttraumatic stress disorder | Biomedical | Longitudinal design | 333 (0%) | SCID | total cholesterol, HDL and LDL cholesterol, triglycerides, and BMI |
| 27 | Park, 2016 | Baroreflex dysfunction and augmented sympathetic nerve responses during mental stress in veterans with post-traumatic stress disorder | Biomedical | Cross sectional study | 28 (11%) | CAPS-IV and PCL-M | MSNA, continuous BP, ECG, inflammatory biomarkers |
| 28 | Ulmer, 2015 | Posttraumatic stress disorder diagnosis is associated with reduced parasympathetic activity during sleep in US veterans and military service members of the Iraq and Afghanistan Wars | Biomedical | Longitudinal design | 62 (13%) | CAPS | BMI |
| 29 | Ulmer, 2015 | Nocturnal blood pressure non-dipping, posttraumatic stress disorder, and sleep quality in women | Public health | Longitudinal design | 124 (100%) | CAPS | Ambulatory BP Monitoring |
| 30 | Gerber, 2015 | Association between mental health burden and coronary artery disease in U.S. women Veterans over 45: A national cross-sectional study | Social science | Cross sectional study | 157195 (100%) | ICD-9 codes in medical records | CVD presence via ICD-9 codes |
| 31 | Ramaswamy, 2015 | Effects of Escitalopram on autonomic function in posttraumatic stress disorder among Veterans of Operations Enduring Freedom and Iraqi Freedom (OEF/OIF) | Social science | Intervention study | 11 (0%) | CAPS-IV | HRV, QTV |
| 32 | Dennis, 2014 | Posttraumatic stress, heart-rate variability, and the mediating role of behavioral health risks | Biomedical | Mediation study | 227 (49%) | CAPS | HRV |
| 33 | Heath, 2013 | Interpersonal violence, PTSD, and inflammation: Potential psychogenic pathways to higher C-reactive protein levels | Biomedical | Cross sectional study | 139 (100%) | PSS | Blood sample for biomarkers of CVD, PHQ-9 |
| 34 | Plantinga, 2013 | Association between posttraumatic stress disorder and inflammation: A twin study | Biomedical | Cross sectional study | 238 twin pairs (0%) | SCID according to DSM-IV | CRP, IL-6, fibrinogen, WBC, CAM-1, and ICAM-1 |
| 35 | Turner, 2013 | Objective evidence of myocardial ischemia in patients with posttraumatic stress disorder | Social science | Longitudinal design | 663 (6%) | CAPS | Exercise treadmill testing, total cholesterol, LDL and HDL cholesterol, CRP |
| 36 | Falger, 1992 | Current posttraumatic stress disorder and cardiovascular disease risk factors in Dutch Resistance Veterans from World War II | Social science | Cross sectional study | 147 (0%) | SCID (DSM-II-R) | Rose Questionnaire  Lifestyles Questionnaire |
| 37 | Fonkoue, 2020 | Symptom severity impacts sympathetic dysregulation and inflammation in post-traumatic stress disorder (PTSD) | Biomedical | Cross sectional study | 70 (27%) | CAPS | BP, HR, HRV, CRP, TNF-𝝰, IL-1B, IL-2, IL-6 |
| 38 | Dennis, 2016 | An investigation of vago-regulatory and health-behavior accounts for increased inflammation in posttraumatic stress disorder | Biomedical | Mediation study | 167 (48%) | CAPS | CRP, TNF-𝝰, IL-10, and TARC/CCL17 |
| 39 | Agorastos, 2013 | Diminished vagal activity and blunted diurnal variation of heart rate dynamics in posttraumatic stress disorder | Biomedical | Cross sectional study | 15 (0%) | CAPS | ECG recording |
| 40 | de Vries, 2015 | Altered one-carbon metabolism in posttraumatic stress disorder | Social science | Cross sectional study | 94 (70%) | SCID (DSM-IV), IES revised | BMI, blood sample for biomarkers of CVD |
| 41 | Weiss, 2011 | Posttraumatic stress disorder is a risk factor for metabolic syndrome in an impoverished urban population | Biomedical | Cross sectional study | 245 (69.6%) | CAPS | BP, weight, height, waist size, blood, and serum samples for biomarkers of CVD |
| 42 | Hughes, 2006 | Posttraumatic stress disorder is associated with attenuated baroreceptor sensitivity among female, but not male, smokers | Social science | Cross sectional study | 130 (46%) | CAPS | BP |
| 43 | Whitworth, 2020 | Cardiorespiratory fitness Is associated with better cardiometabolic health and lower PTSD severity in post-9/11 Veterans | Biomedical | Intervention study | 13 (0%) | CAPS | Height, weight, waist and hip circumferences, and BP, BMI, fasting blood sample |
| 44 | Jeon-Slaughter, 2011 | Heart rate reactivity and current post-traumatic stress disorder when data are missing | Social science | Longitudinal design | 113 (50%) | SCID (DSM IV) | HR, HRV |
| 45 | Teixeira, 2014 | Psychological morbidity and autonomic reactivity to emotional stimulus in parental cancer: A study with adult children caregivers | Biomedical | Intervention study | 78 (77%) | Portuguese version of the IES | I-330-C2 recording device (physiological data), ECG recording, HR |
| 46 | Seligowski, 2020 | Examining the cardiovascular response to fear extinction in a trauma-exposed sample | Social science | Cross sectional study | 51 (100%) | PSS | HR, HRV |
| 47 | Dennis, 2014 | Behavioral health mediators of the link between posttraumatic stress disorder and dyslipidemia | Biomedical | Mediation study | 220 (49%) | CAPS | Serum lipid levels |
| 48 | Sommer, 2019 | Understanding the association between posttraumatic stress disorder characteristics and physical health conditions: A population-based study | Biomedical | Cross sectional study | 1779 (67.5%) | SCID (DSM V) | Self-report of physician diagnosed cardiovascular disease |
| 49 | Sledjeski, 2008 | Does number of lifetime traumas explain the relationship between PTSD and chronic medical conditions? Answers from the National Comorbidity Survey-Replication (NCS-R) | Social science | Cross sectional study | 5692 (43%) | CIDI | Self-report of physician diagnosed CVD |
| 50 | Green, 2016 | Exploring the relationship between posttraumatic stress disorder symptoms and momentary heart rate variability | Biomedical | Longitudinal design | 83 (57%) | Davidson Trauma Scale | Complex demodulation |
| 51 | Bersani, 2016 | Global arginine bioavailability, a marker of nitric oxide synthetic capacity, is decreased in PTSD and correlated with symptom severity and markers of inflammation | Biomedical | Cross sectional study | 121 (0%) | CAPS | CRP, IL-1b, IL-6, TNF-𝝰, IFN, plasma sample concentrations of arginine, ornithine, and citrulline |
| 52 | Von Kanel, 2007 | Evidence for low-grade systemic proinflammatory activity inpatients with posttraumatic stress disorder | Social science | Cross sectional study | 28 (36%) | CAPS | Blood sampling assays |
| 53 | Beckham, 2004 | Cigarette smoking, ambulatory cardiovascular monitoring, and mood in Vietnam Veterans with and without chronic posttraumatic stress disorder | Social science | Longitudinal design | 117 (0%) | Mississippi PTSD Scale, SCID (DSM-III-R) | Ambulatory HR monitoring |
| 54 | Beckham, 2000 | Ambulatory cardiovascular activity in Vietnam combat Veterans with and without posttraumatic stress disorder | Social science | Cross sectional study | 117 (0%) | Mississippi PTSD Scale, SCID (DSM-III-R) | Ambulatory HR monitoring |
| 55 | Sack, 2017 | Intranasal oxytocin reduces provoked symptoms in female patients with posttraumatic stress disorder despite exerting sympathomimetic and positive chronotropic effects in a randomized controlled trial | Biomedical | Intervention study | 35 (100%) | SCID (DSM-IV) | Respiratory frequency, HR, HRV, PEP |
| 56 | Rissling, 2016 | Circadian contrasts in heart rate variability associated with posttraumatic stress disorder symptoms in a young adult cohort | Social science | Longitudinal design | 209 (48%) | Davidson Trauma Scale | ECG, actigraphy, ECG, HRV |
| 57 | Vaccarino, 2013 | Post-traumatic stress disorder and incidence of coronary heart disease | Biomedical | Longitudinal design | 562 (0%) | SCID (DSM-IV) | Myocardial perfusion imaging, myocardial blood flow |
| 58 | Xue, 2012 | Cardiac biomarkers, mortality, and post-traumatic stress disorder in military Veterans | Biomedical | Longitudinal design | 891 (2%) | Self-report of physician diagnosis of PTSD | ECG, CVD medical history, serum samples for CVD biomarkers |
| 59 | Hopper, 2006 | Preliminary evidence of parasympathetic influence on basal heart rate in posttraumatic stress disorder | Biomedical | Intervention study | 59 (85%) | CAPS | ECG |
| 60 | Brass, 1996 | Stroke in former prisoners of war | Biomedical | Longitudinal design | 556 (0%) | Mississippi posttraumatic stress disorder scale | CVD diagnosis from a VA physician |
| 61 | Park, 2019 | Clinical utility of heart rate variability during head-up tilt test in subjects with chronic posttraumatic stress disorder | Social science | Cross sectional study | 139 (8%) | PCL-5 and SCID (DSM-5) | HRV |
| 62 | Shah, 2013 | Posttraumatic stress disorder and impaired autonomic modulation in male twins | Social science | Cross sectional study | 459 (0%) | CAPS | HRV, BP |
| 63 | Clausen, 2016 | Pilot investigation of PTSD, autonomic reactivity, and cardiovascular health in physically healthy combat Veterans | Biomedical | Cross sectional study | 24 (0%) | CAPS | Endothelial function via FMD |
| 64 | Park, 2017 | Baroreflex dysfunction and augmented sympathetic nerve responses during mental stress in veterans with post-traumatic stress disorder | Biomedical | Intervention study | 28 (11%) | CAPS IV | BP, Inflammation |
| 65 | Sawchuk, 2005 | The relationship between post-traumatic stress disorder, depression, and cardiovascular disease in an American Indian tribe | Social science | Cross sectional study | 1414 (51%) | CIDI | Self-report of physician diagnosed CVD |
| 66 | Bullman, 2018 | Comorbidity risks of a cohort of Vietnam Veterans diagnosed with post-traumatic stress disorder | Social science | Longitudinal design | 11, 411 (.13%) | VA physician diagnosis of PTSD according to DSM III, DSM III-R, or DSM IV criteria in VA inpatient and outpatient medical records | Presence of CVD from inpatient and outpatient medical records |
| 67 | Yoo, 2020 | Early onset neurocirculatory response to static handgrip is associated with greater blood pressure variability in women with posttraumatic stress disorder | Biomedical | Cross sectional study | 24 (100%) | CAPS | HR, 24-hour ambulatory BP, BP variability, morning BP |
| 68 | Scherrer, 2020 | PTSD improvement and incident cardiovascular disease in more than 1000 Veterans | Public health | Longitudinal design | 1079 (16.7%) | PCL-C | Incident CVD by ICD-9 codes in medical records |
| 69 | Ulmer, 2013 | Nocturnal blood pressure non-dipping, posttraumatic stress disorder, and sleep quality in women | Public health | Longitudinal design | 148 (100%) | CAPS | Ambulatory BP Monitoring |
| 70 | Sumner, 2017 | Cross-Sectional and longitudinal associations of chronic posttraumatic stress disorder with inflammatory and endothelial function markers in women | Public health | Cross sectional study | 524 (100%) | PTSD screening questionnaire | Plasma biomarkers of CVD |
| 71 | Wingenfeld, 2015 | Effect of current and lifetime posttraumatic stress disorder on 24-hour urinary catecholamines and cortisol: Results from the Mind Your Heart Study | Public health | Cross sectional study | 613 (6%) | CAPS | Self-report medical history |
| 72 | Vidovic, 2011 | Exaggerated platelet reactivity to physiological agonists in war Veterans with posttraumatic stress disorder | Biomedical | Cross sectional study | 27 (0%) | CAPS | Blood sample measurements for CVD biomarkers, height, weight, HR, BP |
| 73 | Jendriƒçko, 2009 | Homocysteine and serum lipids concentration in male war Veterans with posttraumatic stress disorder | Social science | Cross sectional study | 66 (0%) | CAPS | Homocysteine, total cholesterol, HDL and LDL cholesterol, and triglycerides |
| 74 | Vidovic, 2007 | Baseline level of platelet-leukocyte aggregates, platelet CD63 expression, and soluble P-selectin concentration in patients with posttraumatic stress disorder: A pilot study | Biomedical | Cross sectional study | 40 (0%) | ICD-10 code for PTSD | BMI, CVD Risk Factors |
| 75 | Gill, 2010 | Sustained elevation of serum interleukin-6 and relative insensitivity to hydrocortisone differentiates posttraumatic stress disorder with and without depression | Biomedical | Cross sectional study | 32 (47%) | Life Events Checklist, CAPS, SCID (DSM-IV) | Blood serum sample for IL-6 |
| 76 | Newton, 2005 | Ambulatory cardiovascular functioning in healthy postmenopausal women with victimization histories | Social science | Cross sectional study | 39 (100%) | PDS | Ambulatory Readings, BMI |
| 77 | Violanti, 2006 | Posttraumatic stress symptoms and subclinical cardiovascular disease in police officers | Social science | Cross sectional study | 77 (55%) | IES | Ultrasound of the brachial artery, FMD |
| 78 | Richardson, 2009 | Post-traumatic stress disorder and health problems among medically ill Canadian Peacekeeping Veterans | Social science | Cross sectional study | 707 (4.2%) | PCL-M | Self-report of physician-diagnosed CVD |
| 79 | Lee, 2020 | How trauma influences cardiovascular responses to stress: Contributions of posttraumatic stress and cognitive appraisals | Social science | Intervention study | 136 (Not reported) | PCL-5 | BMI, BP, HR |
| 80 | Kibler, 2018 | An extension of the perseverative cognition hypothesis to posttraumatic stress disorder symptomatology: Cardiovascular recovery in relation to posttraumatic stress disorder severity and cognitive appraisals of stress | Social science | Cross sectional study | 50 (100%) | CAPS | Impedance cardiography-derived CO, TPR, HR, BP |
| 81 | Gola, 2013 | Posttraumatic stress disorder is associated with an enhanced spontaneous production of pro-inflammatory cytokines by peripheral blood mononuclear cells | Social science | Cross sectional study | 60 (28%) | CAPS | Plasma cytokine measurements, WBC, PBMC |
| 82 | Britviƒá, 2015 | Comorbidities with posttraumatic stress disorder (PTSD) among combat Veterans: 15 years postwar analysis | Social science | Cross sectional study | 1558 (0%) | SCID (DSM-IV), diagnostic criteria met for PTSD based on the ICD-10, Mississippi Scale for Combat Related Posttraumatic Stress Disorder | CVD presence in medical records validated by 3 trained family physicians |
| 83 | McCubbin, 2016 | Subclinical posttraumatic stress disorder symptoms: Relationships with blood pressure, hostility, and sleep | Biomedical | Cross sectional study | 140 (35%) | PCL | Pittsburgh Sleep Quality Index (sensitive to BP and catecholamine levels in hypertensive patients) |
| 84 | Buckley, 2004 | Twenty-four-hour ambulatory assessment of   heart rate and blood pressure in chronic PTSD and non-PTSD Veterans | Social science | Longitudinal design | 36 (0%) | CAPS | BP monitoring |
| 85 | Spitzer, 2010 | Association of posttraumatic stress disorder with low-grade elevation of C-reactive protein: Evidence from the general population | Social science | Cross sectional study | 3049 (52%) | SCID (DSM-IV) | BP recordings, BMI, Functional Comorbidity Index |
| 86 | Buckley, 2004 | Evaluation of initial posttrauma cardiovascular levels in association with acute PTSD symptoms following a serious motor vehicle accident | Social science | Longitudinal design | 65 (38%) | SCID (DSM-IV) | HR and BP levels at 4 different time points |
| 87 | Forneris, 2004 | Physiological arousal among women Veterans with and without posttraumatic stress disorder | Biomedical | Cross sectional study | 92 (100%) | Trauma Questionnaire, a consensus between two clinicians on PTSD diagnosis | HR, BP, sublingual temperature, BMI |
| 88 | Beckham, 2002 | Magnitude and duration of cardiovascular responses to anger in Vietnam Veterans with and without posttraumatic stress disorder | Social science | Intervention study | 118 (0%) | Self-report of physician diagnosis of PTSD | Continuous BP and HR monitoring |
| 89 | Kibler, 2020 | Body mass index in relation to cardiovascular recovery from psychological stress among trauma-exposed women | Social science | Intervention study | 50 (100%) | CAPS | BMI, ECG |
| 90 | Bukhbinder, 2020 | Increased vascular pathology in older Veterans with a Purple Heart commendation or chronic post-traumatic Stress disorder | Social science | Longitudinal design | 10255 (.8%) | Two records of VA physician diagnosed PTSD | VA medical records of diabetes mellitus, hypertension, hyperlipidemia, and CVD |
| 91 | Rtivedi, 2020 | Mortality among Veterans with major mental illnesses seen in primary care: Results of a national study of Veteran deaths | Biomedical | Longitudinal design | 1763982 (2%) | ICD9 code for PTSD | CVD-related ICD 10 codes in medical records |
| 92 | van den Heuvel, 2020 | Hair cortisol levels in posttraumatic stress disorder and metabolic syndrome | Biomedical | Longitudinal design | 216 (100%) | CAPS | Medical History Questionnaire |
| 93 | Llhua, 2020 | Metabolic syndrome risk in relation to posttraumatic stress disorder among trauma-exposed civilians in Gansu Province, China | Public health | Cross sectional study | 2876 (49%) | PCL-C | Assessment of Metabolic Syndrome |
| 94 | Nichter, 2019 | Physical health burden of PTSD, depression, and their comorbidity in the U.S. Veteran population: Morbidity, functioning, and disability | Social science | Cross sectional study | 2732 (8%) | PCL-S | Medical outcomes SF-8 |
| 95 | Nobles, 2016 | Black-white disparities in the association between posttraumatic stress disorder and chronic illness | Social science | Cross sectional study | 12934 (58%) | CIDI | Self-report of physician diagnosed CVD |
| 96 | Brackbill, 2014 | Chronic physical health consequences of being injured during the terrorist attacks on World Trade Center on September 11, 2001 | Public health | Longitudinal design | 115276 (40%) | 9/11 PTSD Checklist | Self-report of physician diagnosed CVD |
| 97 | Talbot, 2014 | Metabolic risk factors and posttraumatic stress disorder: The role of sleep in young, healthy adults | Biomedical | Longitudinal design | 94 (52%) | CAPS | Lipids, cholesterol, truncal fat, body fat percentage |
| 98 | Brackbill, 2013 | Chronic physical health consequences of being injured during the terrorist attacks on World Trade Center on September 11, 2001 | Social science | Longitudinal design | 14087 (40%) | 9/11 specific PTSD checklist | Self-report of physician diagnosis of CVD |
| 99 | Kemp, 2012 | Depression, comorbid anxiety disorders, and heart rate variability in physically healthy, unmedicated patients: Implications for cardiovascular risk | Social science | Cross sectional study | 73 (68%) | Mini-International Neuropsychiatric Interview | HRV |
| 100 | Pietrzak, 2012 | Physical health conditions associated with posttraumatic stress disorder in U.S. older adults: Results from wave 2 of the National Epidemiologic Survey on Alcohol and Related Conditions | Social science | Cross sectional study | 8533 (55%) | NIAAA Wave 2 Alcohol Use Disorder and Associated Disabilities Interview Schedule DSM-IV | SF-12-Item Health Survey |
| 101 | Glaesmer, 2011 | The association of traumatic experiences and posttraumatic stress disorder with physical morbidity in old age: A German population-based study | Social science | Cross sectional study | 1456 (52%) | PDS | Self-report medical history questionnaire |
| 102 | Pietrzak, 2011 | Medical comorbidity of full and partial posttraumatic stress disorder in US adults: Results from wave 2 of the National Epidemiologic Survey on Alcohol and Related Conditions | Biomedical | Cross sectional study | 31650 (52%) | Self-report of physician diagnosis of PTSD | Self-report of physician diagnosis of CVD |
| 103 | David, 2004 | Comparison of comorbid physical illnesses among Veterans with PTSD and Veterans with alcohol dependence | Social science | Cross sectional study | 93 (0%) | SCID (DSM III and IV) | Blood cell count, glucose, triglycerides, and cholesterol, CVD diagnosis by board-certified internal medicine specialist |
| 104 | Song, 2019 | Stress related disorders and risk of cardiovascular disease: Population based, sibling-controlled cohort study | Biomedical | Longitudinal design | 136637 (62.5%) | PTSD ICD 9 code | CVD related ICD codes in medical records |
| 105 | Ferretti, 2019 | Post-traumatic stress disorder in Italy: A comprehensive evaluation of all the ICD comorbidities and gender-related differences | Biomedical | Cross sectional study | 84 (43%) | CAPS | Self-report of CVD |
| 106 | Gibson, 2018 | Gender differences in cardiovascular risk related to diabetes and posttraumatic stress disorder | Social science | Longitudinal design | 2789264 (6%) | At least one medical record of PTSD diagnosis during the baseline period | Incidence of CVD during follow-up period |
| 107 | Kibler, 2018 | Cardiovascular risks in relation to posttraumatic stress severity among young trauma-exposed women | Social science | Cross sectional study | 54 (100%) | SCID (DSM-IV) | Lipid levels, BMI, resting BP |
| 108 | McLeay, 2017 | Physical comorbidities of post-traumatic stress disorder in Australian Vietnam War Veterans | Biomedical | Cross sectional study | 298 (0%) | CAPS-5 | Self-report of physician diagnosed CVD, ECG, CAC |
| 109 | Scherrer, 2019 | Comorbid conditions explain the association between posttraumatic stress disorder and incident cardiovascular disease | Public health | Longitudinal design | 4178 (13%) | Two documented visits with an ICD-9 code for PTSD | Incident CVD via ICD-9 codes in medical records |
| 110 | Burg, 2017 | Risk for incident hypertension associated with PTSD in military Veterans, and the effect of PTSD treatment | Social science | Longitudinal design | 194319 (15%) | Two or more outpatient medical records of PTSD or one or more inpatient record | A medical diagnosis of hypertension, a new prescription for anti-hypertensive medication, and/or a clinic BP reading in the hypertensive range (greater or equal to 140mmHg/90mmHg, systolic/diastolic). |
| 111 | Farr, 2015 | Posttraumatic stress disorder, alone or additively with early life adversity, is associated with obesity and cardiometabolic risk | Biomedical | Cross sectional and longitudinal design | 158 (52.5%) | Evaluation for Lifetime Stressors interview; SCID (DSM-IV-R); UCLA PTSD scale; Adult Attachment Interview | BMI, systolic and diastolic BP, fat mass, waist circumference, CRP, fasting blood glucose, total cholesterol |
| 112 | Howard, 2018 | Associations of initial injury severity and posttraumatic stress disorder diagnoses with long-term hypertension risk after combat injury | Biomedical | Longitudinal design | 3846 (1.7%) | ICD-9 code for PTSD in medical record | CVD-related ICD-9 code in medical records |
| 113 | Sawchuk, 2005 | The relationship between post-traumatic stress disorder, depression, and cardiovascular disease in an American Indian tribe | Social science | Cross sectional study | 1414 (51%) | CIDI | Self-report of CVD and CVD risk factors |
| 114 | Goldstein, 2017 | Characteristics and health care preferences associated with cardiovascular disease risk among women Veterans | Social science | Cross sectional study | 3587 (100%) | Breslau's 7-item screen for posttraumatic stress disorder | Self-report of physician diagnosed hypertension and/or diabetes and smoking |
| 115 | Nevell, 2014 | Elevated systemic expression of ER stress related genesis associated with stress-related mental disorders in the Detroit Neighborhood Health Study | Biomedical | Cross sectional study | 86 (61%) | PCL | Major indicators of ER stress response (BiP, EDEM1, CHOP, and XBP1) |
| 116 | Ferretti, 2019 | A comparison of physical comorbidities in patients with posttraumatic stress disorder developed after a terrorist attack or other traumatic event | Biomedical | Cross sectional study | 84 (43%) | Mini International Neuropsychiatric Interview, CAPS, and the Davidson Trauma Scale | Self-report of CVD |
| 117 | Moazen-Zadeh, 2016 | Increased blood pressures in Veterans with post-traumatic stress disorder: A case-control study | Biomedical | Cross sectional study | 100 (0%) | Diagnostic interview from military psychiatrist (DSM-IV-TR) | Brachial systolic and diastolic BP |
| 118 | Sareen, 2007 | Physical and mental comorbidity, disability, and suicidal behavior associated with posttraumatic stress disorder in a large community sample | Social science (psychology, social work, sociology etc.) | Cross sectional study | 36984 (55%) | Self-report of PTSD diagnosed by a clinician in the past 6 months | Self-report of physician diagnosed CVD |
| 119 | Jordan, 2013 | Cardiovascular disease hospitalizations in relation to exposure to the September 11, 2001, World Trade Center disaster and posttraumatic stress disorder | Biomedical | Longitudinal design | 46346 (40%) | PCL | CVD-related hospitalization incidence |
| 120 | Beristianos, 2014 | PTSD and risk of incident cardiovascular disease in aging Veterans | Public health | Longitudinal design | 138341 (4.1%) | ICD-9-CM code for PTSD | CVD or cerebrovascular disease ICD-9 codes |
| 121 | Dennis, 2017 | Trauma and autonomic dysregulation: episodic- versus systemic-negative affect underlying cardiovascular risk in posttraumatic stress disorder | Biomedical | Mediation study | 197 (51%) | CAPS | ECG, HR and HRV monitoring, FMD, hyperemic flow |
| 122 | Kang, 2006 | Risk of selected cardiovascular diseases and posttraumatic stress disorder among former World War II prisoners of war | Social science | Longitudinal design | 29170 (0%) | ICD-9 code for PTSD in medical record | CVD ICD-9 codes in medical record |
| 123 | van Dammen, 2019 | Childhood adversity and women's cardiometabolic health in adulthood: associations with health behaviors, psychological distress, mood symptoms, and personality | Biomedical | Cross sectional study | 115 (100%) | Primary care PTSD screen | Height and weight, hip circumference, BP, and blood samples for biomarkers of metabolic syndrome |
| 124 | Sumner, 2016 | Associations of trauma exposure and Posttraumatic stress symptoms with venous thromboembolism over 22 years in women | Biomedical | Cross sectional study | 49, 296 (100%) | Brief Trauma Questionnaire, Short screening scale for PTSD (DSM-IV) | Self-report of venous thromboembolism |
| 125 | Grenon, 2015 | Posttraumatic stress disorder is associated with worse endothelial function among Veterans | Biomedical | Cross sectional study | 214 (6%) | PCL | Brachial artery FMD |
| 126 | Caska, 2014 | Posttraumatic stress disorder and responses to couple conflict: Implications for cardiovascular risk | Social science | Cross sectional study | 64 (50%) | CAPS, PCL-M, PCL-C | BP and HR |
| 127 | Edmondson, 2018 | The association of PTSD with clinic and ambulatory blood pressure in healthy adults | Biomedical | Cross sectional study | 440 (62%) | PCL-C | Mean of nine BP readings, separate readings for systolic and diastolic BP |
| 128 | Flood, 2010 | Prospective study of externalizing and internalizing subtypes of posttraumatic stress disorder and their relationship to mortality among Vietnam veterans | Social science | Cross sectional study | 5248 (0%) | CAPS | CVD-related causes of death in death records |
| 129 | Goetz, 2014 | Posttraumatic stress disorder, combat exposure, and carotid intima-media thickness in male twins | Public health | Cross sectional study | 465 (0%) | SCID (DSM IV), Combat Exposure Scale | CIMT |
| 130 | Blessing, 2016 | Biological predictors of insulin resistance associated with posttraumatic stress disorder in young military veterans | Biomedical | Cross sectional study | 166 (0%) | SCID (DSM-IV) | Self-report of physician diagnosis of CVD |
| 131 | de Vris, 2017 | Plasma lipoproteins in posttraumatic stress disorder patients compared to healthy controls and their associations with the HPA- and HPT-axis | Biomedical | Cross sectional study | 94 (38%) | SCID | Waist circumference, BMI, blood samples for biomarkers of CVD |
| 132 | Rooks, 2012 | Early trauma and Inflammation: Role of familial factors in a study of twins | Biomedical | Cross sectional study | 241 twin pairs (0%) | SCID (DSM-IV) | CRP, IL-6, and Self-report of CVD risk factors and disease |
| 133 | Mellman, 2009 | Posttraumatic stress disorder and nocturnal blood pressure dipping in young adult African Americans | Biomedical | Cross sectional study | 30 (60%) | CAPS | 24-hour ambulatory BP |
| 134 | Oddone, 2015 | Orthostatic hypotension in young adults with and without posttraumatic stress disorder | Social science | Cross sectional study | 222 (49%) | CAPS | Supine and standing systolic BP and diastolic BP, BMI |
| 135 | Fetzner, 2012 | Similarities in specific physical health disorder prevalence among formerly deployed Canadian forces veterans with full and subsyndromal PTSD | Social science | Cross sectional study | 990 (2.3%) | PCL-M | Self-report of physician diagnosed CVD |
| 136 | von Kanel, 2006 | Altered blood coagulation in patients with posttraumatic stress disorder | Biomedical | Cross sectional study | 29 (34%) | German version of CAPS | Blood sampling for biomarkers of CVD |
| 137 | Sperling, 2012 | Somatic diseases in child survivors of the Holocaust with posttraumatic stress disorder | Social science | Longitudinal design | 120 (47%) | PTSD ICD-10 code | CVD related ICD-9 codes |
| 138 | Paulus, 2013 | The Impact of posttraumatic stress disorder on blood pressure and heart rate in a Veteran population | Social science | Longitudinal design | 186 (0%) | PTSD diagnosis in healthcare system | BP and HR |
| 139 | Lin, 2019 | Risk of incident hypertension, diabetes, and dyslipidemia after first posttraumatic stress disorder diagnosis: A nationwide cohort study in Taiwan | Biomedical | Longitudinal design | 4765 (50%) | PTSD ICD-9 code, board certified psychiatrist diagnosis | Metabolic Syndrome Diagnosis |
| 140 | Kibler, 2009 | Hypertension in relation to posttraumatic stress disorder and depression in the US National Comorbidity Survey | Social science | Cross sectional study | 5877 (55%) | CIDI | Self-report health conditions and CVD |
| 141 | Meyer, 2016 | Posttraumatic stress disorder (PTSD) patients exhibit a blunted parasympathetic response to an emotional stressor | Biomedical | Cross sectional study | 89 (100%) | SIDES (German version) | HR, ECG, beat-to-beat BP, arterial BP, and cardiac index |
| 142 | Khazai, 2011 | Abnormal ECG patterns in chronic post-war PTSD patients: A pilot study | Social science | Cross sectional study | 82 (11%) | SCID | 12-lead ECG |
| 143 | von Kanel, 2008 | Measures of endothelial dysfunction in plasma of patients with posttraumatic stress disorder | Social science | Cross sectional study | 28 (36%) | German version of CAPS | Physician Diagnosis of high blood pressure, glucose levels, hypercholesterolemia, BMI, BP |
| 144 | Dirkzwager, 2007 | Disaster-related posttraumatic stress disorder and physical health | Biomedical | Longitudinal design | 896 (50%) | Self-Rating Scale for Post-Traumatic Stress Disorder | CVD diagnosis via family practitioner's data (compatible with ICD-10) |
| 145 | Kinzie, 2008 | High prevalence rates of diabetes and hypertension among refugee psychiatric patients | Social science | Cross sectional study | 459 (68%) | Psychiatric interview (DSM-IV) | Hypertension diagnosis |
| 146 | Vidal, 2018 | Co-occurrence of posttraumatic stress disorder and cardiovascular disease among ethnic/racial groups in the United States | Biomedical | Cross sectional study | 9842 (51%) | CIDI | Self-report of physician diagnosis of CVD or hypertension. |
| 147 | Tsai, 2017 | Exploring the link between posttraumatic stress disorder and inflammation-related medical conditions: An epidemiological examination | Biomedical | Cross sectional study | 1527 (58%) | PTSD diagnosis in parent study | Self-report of medical conditions associated with inflammation and heart disease |
| 148 | Rosman, 2019 | Posttraumatic stress disorder and risk for stroke in young and middle-Aged adults: A 13-year cohort study | Biomedical | Longitudinal design | 987855 (12.2%) | ICD-9 code for PTSD listed before the index stroke | TIA and stroke incidence. |
| 149 | Remch, 2018 | Post-traumatic stress disorder and cardiovascular diseases: A cohort study of men and women Involved in cleaning the debris of the World Trade Center Complex | Biomedical | Longitudinal design | 6481 (16%) | PCL-C | BP, body weight, blood lipids, BMI |
| 150 | Yu, 2018 | Risk of stroke among survivors of the September 11, 2001, World Trade Center Disaster | Biomedical | Cross sectional study | 42527 (39%) | PCL | Physician confirmation of stroke |
| 151 | Ahmadi, 2018 | The long-term clinical outcome of posttraumatic stress disorder with Impaired coronary distensibility | Biomedical | Longitudinal design | 246 (30%) | CAPS, PCL-M | CDI, CAD, MACE |
| 152 | Walczewska, 2011 | Stiffness of large arteries and cardiovascular risk in patients with post-traumatic stress disorder | Biomedical | Cross sectional study | 150 (50%) | Psychiatric Interview (DSM-IV) | BP, BMI, glucose levels |
| 153 | Pizur-Barnekow, 2010 | Maternal health after the birth of a medically complex infant: Setting the context for evaluation of co-occupational performance | Social science | Longitudinal design | 10 (100%) | Perinatal posttraumatic Stress Disorder Questionnaire | BMI, BP, Weight, ECG |
| 154 | Zucker, 2009 | The effects of respiratory sinus arrhythmia biofeedback on heart rate variability and posttraumatic stress disorder symptoms: A pilot study | Social science | Intervention study | 38 (45%) | DAPS | HRV amplitude |
| 155 | Kubzansky, 2009 | A prospective study of posttraumatic stress disorder symptoms and coronary heart disease in women | Public health | Longitudinal design | 1059 (100%) | NIMH DIS | BMI, presence of hypertension and diabetes |
| 156 | Kulenovic, 2008 | Changes in plasma lipid concentrations and risk of coronary artery disease in army Veterans suffering from chronic posttraumatic stress disorder | Biomedical | Cross sectional study | 100 (0%) | IES-90-R, Folkman-Lazarus Coping Strategies Questionnaire | Plasma lipid levels |
| 157 | Boscarino, 2008 | A prospective study of PTSD and early-age heart disease, mortality among Vietnam Veterans: Implications for surveillance, and prevention | Biomedical | Longitudinal design | 4328 (0%) | DSM-III measures for PTSD and K-PTSD | BP, resting ECG |
| 158 | Kubzansky, 2007 | Prospective study of posttraumatic stress disorder symptoms and coronary heart disease in the Normative Aging Study | Biomedical | Longitudinal design | 2280 (0%) | Mississippi Scale for Combat Related PTSD, MMPI-2 PK | Physical exam of biochemical values, serum cholesterol, weight, and BP |
| 159 | Gilsanz, 2017 | Posttraumatic stress disorder symptom duration and remission in relation to cardiovascular disease risk among a large cohort of women | Social science | Longitudinal design | 49859 (100%) | Brief Trauma Questionnaire, 7-item Short Screening Scale for DSM-IV | Self-report of physician diagnosis of CVD at each biennial interview |
| 160 | Gradus, 2015 | Associations between stress disorders and cardiovascular disease events in the Danish population | Social science | Longitudinal design | 69579 (62%) | ICD-9 code for PTSD | CVD incidence via ICD-9 codes |
| 161 | Rooks, 2015 | Long-term consequences of early trauma on coronary heart disease: Role of familial factors | Social science | Cross sectional study | 562 Twin pairs (0%) | SCID | BMI, measure of myocardial blood flow |
| 162 | Sumner, 2015 | Trauma exposure and posttraumatic stress disorder symptoms predict onset of cardiovascular events in women | Biomedical | Cross sectional study | 54282 (100%) | Brief Trauma Questionnaire and PTSD Screen | Physician diagnosed CVD |
| 163 | Roy, 2015 | Posttraumatic stress disorder and incident heart failure among a community-based sample of US veterans | Public health | Longitudinal design | 8248 (4%) | ICD-9 code for PTSD | CVD via ICD-9 codes |
| 164 | Chen, 2015 | Risk of stroke among patients with post-traumatic stress disorder: Nationwide longitudinal study | Social science | Longitudinal design | 5217 (79%) | Board certified psychiatrist diagnosis of PTSD at time of enrollment | Diagnoses of any type of stroke by neurologists, neurosurgeons, and emergency room physicians from brain image examinations |
| 165 | Hefner, 2015 | Recurrent Tako-Tsubo cardiomyopathy (TTC) in a pre-menopausal woman: late sequelae of atraumatic event? | Biomedical | Cross sectional study | 1 (100%) | Chronic post-traumatic stress syndrome diagnosis | Takotsubo cardiomyopathy diagnosis |
| 166 | An, 2015 | Psychological strains, salivary biomarkers, and risks for coronary heart disease among hurricane survivors | Biomedical | Cross sectional study | 19 (89%) | PCL-C | BP average after 3 measurements, cholesterol, and blood glucose levels,  salivary concentrations of IL-1B, IL-6, IL-10, and MCP-1 |
| 167 | Crum-Cianflone, 2014 | Impact of combat deployment and posttraumatic stress disorder on newly reported coronary heart disease among US active duty and reserve forces | Social science | Longitudinal design | 60025 (27%) | PCL-C | Self-reported absence of a physician diagnosis of CVD at and before the baseline survey and the presence of CVD at follow up (excluding chest pain and angina) |
| 168 | Scott, 2013 | Associations between DSM-IV mental disorders and subsequent heart disease onset: Beyond depression | Public health | Longitudinal design | 52095 (Not reported) | CIDI | Self-report of physician diagnosed CVD |
| 169 | Turner, 2013 | Objective evidence of myocardial ischemia in patients with posttraumatic stress disorder | Public health | Cross sectional study | 663 (6%) | CAPS | Standardized exercise treadmill test, myocardial ischemia, LDL and HDL cholesterol |
| 170 | Wagner, 2013 | Trauma, healthcare access, and health outcomes among Southeast Asian refugees in Connecticut | Biomedical | Cross sectional study | 229 (54%) | Harvard Trauma Questionnaire | Self-report of physician diagnosed health conditions |
| 171 | Jordan, 2011 | Heart disease among adults exposed to the September 11, 2001, World Trade Center disaster: Results from the World Trade Center Health Registry | Biomedical | Longitudinal design | 1162 (33%) | PCL | Self-report of physician diagnosis of angina, heart attack, or any other heart condition |
| 172 | Chaudieu, 2011 | Late-life health consequences of exposure to trauma in a general elderly population: The mediating role of reexperiencing posttraumatic symptoms | Social science | Longitudinal design | 1662 (60%) | Watson's PTSD Inventory and the Mini International Neuropsychiatric Interview | Blood samples for biomarkers of CVD and health interview with a general practitioner |
| 173 | Ahmadi, 2011 | Post-traumatic stress disorder, coronary atherosclerosis, and mortality | Biomedical | Cross sectional study | 637 (12.2%) | CAPS and PCL-M | CAC, BP, total cholesterol, LDL and HDL cholesterol, triglycerides, Framingham risk score |
| 174 | Jin, 2011 | Increased Framingham 10-year risk of coronary heart disease in middle-aged and older patients with psychotic symptoms | Social science | Longitudinal design | 179 (32%) | SCID (DSM-IV) | 10-year CHD risk via LDL and HDL cholesterol, BP, diabetes, smoking status incidence |
| 175 | Solter, 2002 | Elevated serum lipids in veterans with combat-related chronic posttraumatic stress disorder | Biomedical | Cross sectional study | 195 (0%) | SCID (DSM-III), Watson's PTSD inventory | Blood samples of biomarkers of CVD, BP |
| 176 | Boscarino, 1999 | Electrocardiogram abnormalities among men with stress-related psychiatric disorders: Implications for coronary heart disease and clinical research | Biomedical | Longitudinal design | 48,513 (0%) | DIS | ECG |
